# Meningocele in a Congolese Female with Beckwith-Wiedemann Phenotype

**DOI:** 10.1155/2014/989425

**Published:** 2014-12-28

**Authors:** Sébastien Mbuyi-Musanzayi, Toni Lubala Kasole, Aimé Lumaka, Tony Kayembe Kitenge, Leon Kabamba Ngombe, Prosper Kalenga Muenze, Prosper Lukusa Tshilobo, François Tshilombo Katombe, Célestin Banza Lubaba Nkulu, Koenraad Devriendt

**Affiliations:** ^1^Department of Surgery, University Hospital, University of Lubumbashi, P.O. Box 1825, Lubumbashi, Democratic Republic of the Congo; ^2^Center for Human Genetics, Faculty of Medicine, University of Lubumbashi, P.O. Box 1825, Lubumbashi, Democratic Republic of the Congo; ^3^Department of Pediatrics, University Hospital, University of Lubumbashi, P.O. Box 1825, Lubumbashi, Democratic Republic of the Congo; ^4^Department of Pediatrics, University Hospital, University of Kinshasa, P.O. Box 123, Kin XI, Kinshasa, Democratic Republic of the Congo; ^5^Center for Human Genetics, University Hospital, KU Leuven, Campus Gasthuisberg, Herestraat 49, P.O. Box 602, 3000 Leuven, Belgium; ^6^Unit of Toxicology and Environment, School of Public Health, University Hospital, University of Lubumbashi, P.O. Box 1825, Lubumbashi, Democratic Republic of the Congo; ^7^Department of Gynecology, University Hospital, University of Lubumbashi, P.O. Box 1825, Lubumbashi, Democratic Republic of the Congo

## Abstract

Beckwith-Wiedemann syndrome (BWS) is a rare congenital syndrome characterized by an overgrowth, macroglossia, exomphalos, and predisposition to embryonal tumors. Central nervous abnormalities associated with BWS are rare. We describe a one-day-old Congolese female who presented meningocele associated with BWS phenotype.

## 1. Introduction

Beckwith-Wiedemann syndrome (BWS) is a rare congenital disorder with an incidence of one in 13.700 live births [1, 2]. Initially, its designation was EMG (exomphalos-macroglossia-gigantism syndrome) and was characterized by a triad of an overgrowth, macroglossia, and exomphalos or umbilical hernia [3]. Other features were observed being associated with the triad, such as umbilical hernia, organomegaly (liver, spleen, or kidneys), neonatal hypoglycemia, minor ear anomalies, nevus flammeus, cleft palate, or embryonal tumor development [4, 5]. The clinical features of BWS are variable, and it is accepted that the diagnosis can be established if three major diagnostic findings are present [6]. Its etiology is heterogeneous, arising from dysregulation of one or both imprinting control regions (IC) and/or imprinted growth regulatory genes of the chromosome 11p15.5 [7]. BWS occurs with the same frequency in male and female [8]. Central nervous system anomalies are rare in BWS, but as far as we know not a single case has been described presenting meningocele. Here we present a female newborn who presented meningocele associated with the BWS phenotype.

## 2. Case Report

**Figure 1:**
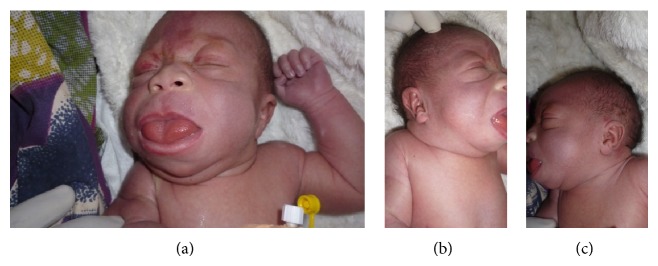
Facial signs: note (a) macroglossia and nevus flammeus on the face; (b) crumped helix on her right ear; (c) ear crease on the left lobe.

**Figure 2:**
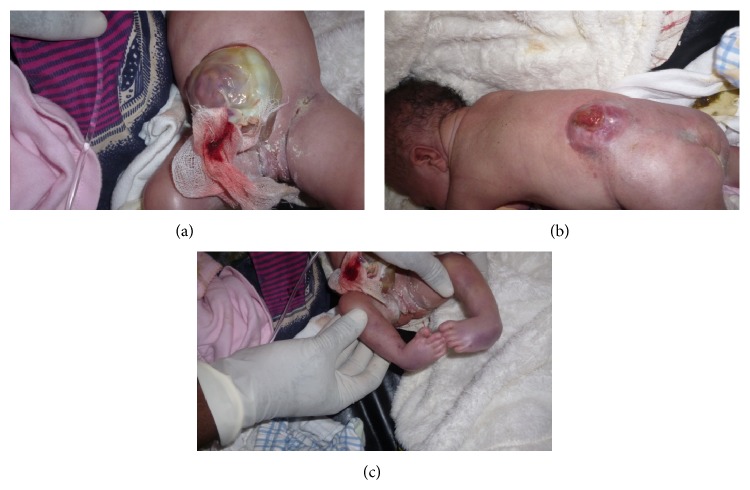
Other features: note (a) exomphalos, (b) meningocele, and (c) bilateral club feet.

The index case is a one-day-old female, born at 39 weeks of gestation via normal spontaneous vaginal delivery with a birth weight of 4400 g (+4 SD, CDC growth charts). Her mother was 27 years, her father was 30 years old, and both were healthy and unrelated. Family history was marked by a primary infertility for 10 years. The mother had many gynecological consultations and medical treatment that she ignored during the last two years. During gestation she had a urinary infection treated with amoxicillin during the first trimester and treatment of threatened miscarriage. She took clay during the entire pregnancy. She received vaccination during the second trimester of gestation. Two prenatal ultrasound scans were performed, one during the first trimester and the second during the second trimester. Both did not show any anomaly. At birth on clinical examination, we observed macroglossia with protruding tongue, nevus flammeus on her face (Figure 1(a)). She presented crumped helix on her right ear and ear creases on the left lobe (Figures 1(b) and 1(c)). She had an omphalocele containing intestines (Figure 2(a)), a lumbosacral meningocele, and bilateral club feet (Figures 2(b) and 2(c)). She developed acute respiratory distress and died within six hours after birth.

## 3. Discussion

We present a newborn female, who presented an unusual association, meningocele associated with BWS phenotype. The diagnosis of BWS was based on the classical clinic triad of overgrowth, macroglossia, and exomphalos, associated with some additional minor features such as ear anomalies and frontal hemangioma. The diagnosis could not be confirmed by genetic studies, since the child died prematurely, before DNA could be obtained. The distinctive feature in the present case is lumbosacral meningocele. Central nervous abnormalities associated with BWS are rare, mostly involving the brain (reviewed in Table 1). It is not excluded that this represents a chance association of two pathogenetically unrelated conditions, and the lack of genetic testing is a weakness of this report. Alternatively, given the previous reports of CNS anomalies in other cases with BWS, it is not excluded that the underlying genetic cause of BWS may also predispose to brain malformations (including neural tube defects), especially when imprinting defect involves the imprinting domain 2 at chromosome 11p15.5 [16].

## Figures and Tables

**Table 1:** Central nervous features associated with BWS.

Study	Worth et al. [9]	Yamada et al. [10]	Tubbs and Oakes [11]	Russo et al. [12]	Broekman et al. [13]	Kent et al. [14]	Bui et al. [15]	Gardiner et al. [16]	This report
Year	1999	1999	2005	2006	2008	2008	2009	2012	2014
Brain abnormalities									
Abnormal cerebellar vermis	—	—	—	+^*^	—	—	—	—	—
Arteriovenous malformations	—	+	—	—	—	—	—	—	—
Blake's pouch cyst	—	—	—	—	—	—	—	2/7	—
Chiari malformation	—	—	+	—	—	—	—	—	—
Dandy-Walker malformation	—	—	—	—	—	—	—	3/7	—
Dandy-Walker variant	—	—	—	—	—	—	—	4/7	—
Encephalocele (nasal)	—	—	—	—	+	—	—	—	—
Hydrocephalus	—	—	—	—	—	+	—	—	—
Posterior fossa structures abnormalities	—	—	—	—	—	—	+	—	—
Schizencephaly	+	—	—	—	—	—	—	—	—
Meningocele	—	—	—	—	—	—	—	—	+

^*^In this case, the associated terminal deletion of chromosome 4p may possibly explain the central nervous system malformation.
